# Y-box binding protein YBX1 and its correlated genes as biomarkers for poor outcomes in patients with breast cancer

**DOI:** 10.18632/oncotarget.26469

**Published:** 2018-12-14

**Authors:** Tomohiro Shibata, Eriko Tokunaga, Satoshi Hattori, Kosuke Watari, Yuichi Murakami, Nami Yamashita, Eiji Oki, Junji Itou, Masakazu Toi, Yoshihiko Maehara, Michihiko Kuwano, Mayumi Ono

**Affiliations:** ^1^ Department of Pharmaceutical Oncology, Graduate School of Pharmaceutical Sciences, Kyushu University, Fukuoka, Japan; ^2^ National Hospital Organization Kyushu Cancer Center, Fukuoka, Japan; ^3^ Department of Integrated Medicine, Biomedical Statistics, Osaka University Graduate School of Medicine, Osaka, Japan; ^4^ Cancer Translational Research Center, St. Mary's Institute of Health Sciences, Kurume, Japan; ^5^ Department of Surgery and Science, Graduate School of Medical Sciences, Kyushu University, Fukuoka, Japan; ^6^ Department of Breast Surgery, Graduate School of Medicine, Kyoto University, Kyoto, Japan; ^7^ Kyushu Central Hospital of the Mutual Aid Association of Public School Teachers, Fukuoka, Japan

**Keywords:** YBX1, ER, breast cancer, predictive biomarker, antiestrogen resistance

## Abstract

The enhanced expression of the Y-box binding protein YBX1 is consistently correlated with poor outcomes or reduced survival of breast cancer patients. However, the mechanism underlying the association between increased YBX1 expression and poor outcomes has yet to be revealed. We searched a database for the top 500 genes that are positively or negatively correlated with YBX1 and with ESR1 in breast cancer patients. We further examined the association between YBX1-correlated genes and breast cancer outcomes in patients at Kyushu University Hospital. More than 60% of genes that are positively correlated with YBX1 are also negatively correlated with ESR1. The enhanced expression levels of the top 20 positively correlated genes mostly predict negative outcomes, while the enhanced expression levels of the top 20 negatively correlated genes mostly predict positive outcomes. Furthermore, in breast cancer patients at Kyushu University Hospital, the expression levels of YBX1 and YBX1-positively correlated genes were significantly higher and the expression levels of genes negatively correlated with YBX1 were significantly lower in patients who relapsed after their primary surgery than in those who did not relapse. The expression of YBX1 together with the expression of its positively or negatively correlated genes may help to predict outcomes as well as resistance to endocrine therapies in breast cancer patients. Determining the expression of YBX1 and its closely correlated genes will contribute to the development of precision therapeutics for breast cancer.

## INTRODUCTION

Growth in approximately 70% of breast cancers depends on estrogen receptor (ER) expression and its related signal transduction pathway, while growth in approximately 20% of breast cancers depends on the HER2/ERBB2 status [1, 2]. Endocrine therapies have provided significant benefits for patients with ER-positive breast cancers [3, 4], and HER2-targeted therapeutics have similarly provided benefits for patients with HER2-positive breast cancers [2, 5]. However, the generation of refractory tumors that are resistant to these therapies is a serious obstacle to the further improvement of treatments for breast cancer patients [6, 7]. Identifying other genes or molecules involved in the progression of breast cancer is thus expected to contribute to the more precise prediction of the outcomes of and treatments for refractory tumors [8].

Y-box binding protein (YBX1), a DNA/RNA binding protein containing an evolutionarily conserved cold-shock domain, regulates transcription, translation, DNA damage repair, and other biological processes that occur in both the nucleus and cytoplasm [9–11]. Cytoplasmic YBX1 regulates mRNA stability and translation [12], and nuclear YBX1 plays a key role in transcriptional regulation via the Y-box binding site (inverted CCAAT box) [13, 14]. YBX1 plays a major role in the host’s defense mechanisms against environmental cytotoxic stimuli as well as in the growth, survival, and drug resistance of tumor cells [10, 11, 15].

Because YBX1 was identified as an oncoprotein in breast cancer via transgenic knock-in animal models of various types of breast cancer [16], it is therefore expected that YBX1 may play a specific and essential role in the tumorigenesis and malignant progression of breast cancer. YBX1 converts human mammary epithelial cells into breast cancer cells capable of anchorage-independent growth [17]. It also promotes cell growth and increases the expression of HER2 as well as other genes that mediate the cell cycle, cell proliferation, and drug resistance in breast cancer cells [18–23]. YBX1 decreases the response to tamoxifen and fulvestrant in ER-positive breast cancer through the downregulation of the ER protein [24], and antiestrogen resistance is also mediated through YBX1 activation by FGFR2 [25]. We previously demonstrated that the resistance of breast cancer to antiestrogens is mediated through both increased proteasomal ER degradation and increased transcriptional activation of HER2 by YBX1 [26] and that YBX1 negatively and positively correlates with ER and HER2 expression, respectively, in clinical specimens from breast cancer patients as well as in breast cancer cells *in vitro* and *in vivo* [21, 26]. However, the mechanism underlying the predictive ability of YBX1 for a poor prognosis is still not well understood.

In the present study, we searched a database for the top 500 genes that are positively and negatively correlated with YBX1 in breast cancer. Most of the genes that were negatively correlated with YBX1 overlapped with genes that were positively associated with ESR1, and most of the genes that were positively correlated with YBX1 overlapped with genes that were negatively associated with ESR1. However, the clinical significance of YBX1 in breast cancer should be discussed in the context of genes facilitating ER-dependent or ER-independent growth and survival.

## RESULTS

### Genes that are positively or negatively correlated with YBX1 predict the outcomes of breast cancer patients

**Figure 1:**
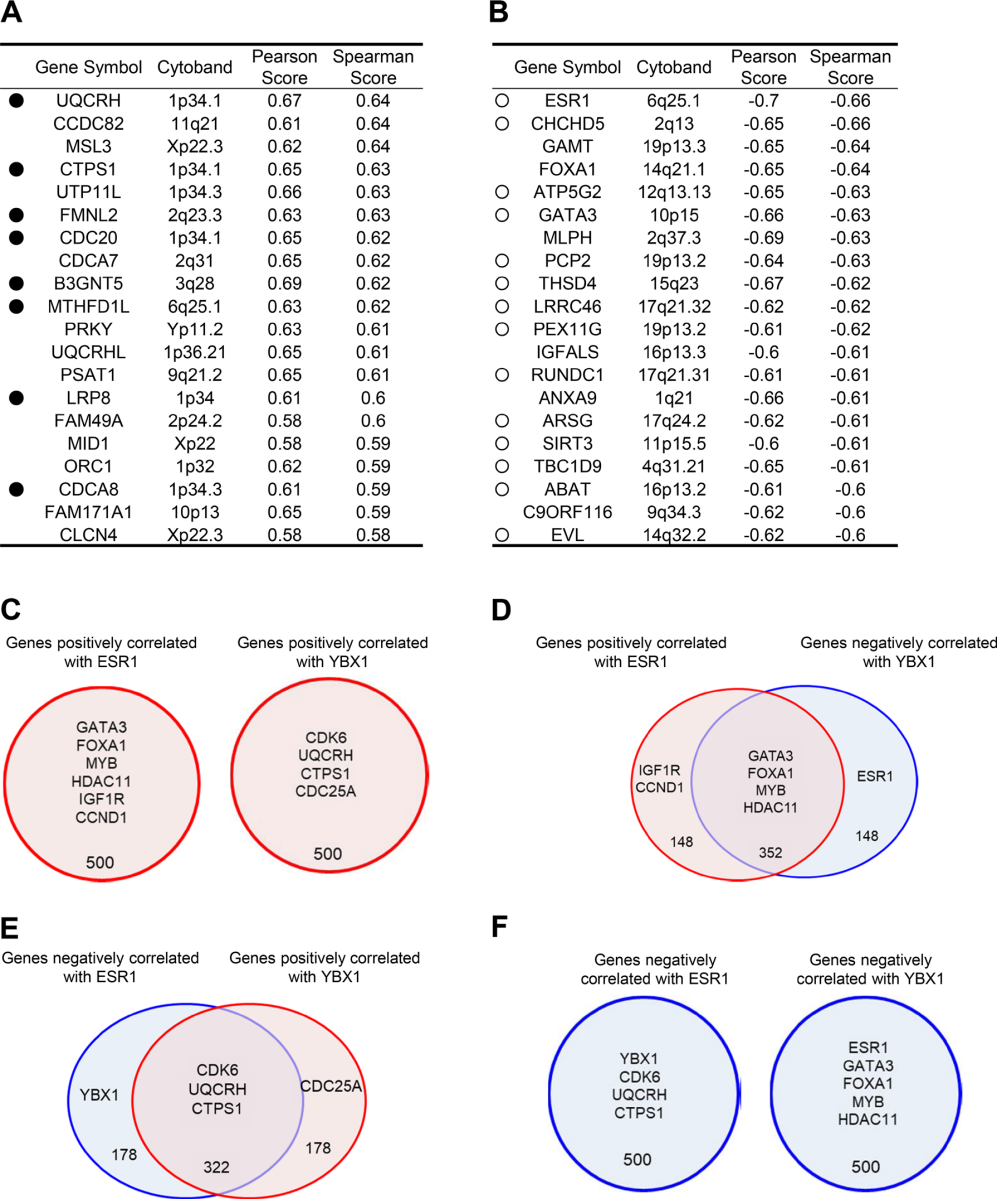
Genes correlated with YBX1 are associated with prognostic factors (**A**, **B**) Lists of the top 20 genes positively (A) or negatively (B) correlated with YBX1. RNA-seq expression data from human invasive breast cancer samples (*n* = 825) were analyzed for their association with YBX1. The genes are listed in order of Spearman’s rank positive (A) or negative (B) correlation coefficients for the associations between YBX1 and the other genes. Black dots indicate poorly prognostic genes (see also Supplementary Figure 1). White dots indicate good prognostic genes (see also Supplementary Figure 2). (**C**) A Venn diagram showing the overlap of the top 500 genes that are positively correlated with ESR1 and the top 500 genes that are positively correlated with YBX1. The top 500 genes that are positively correlated with ESR1 showed no overlap with the top 500 genes that are positively correlated with YBX1. (**D**) A Venn diagram showing the overlap of the top 500 genes that are positively correlated with ESR1 and the top 500 genes that are negatively correlated with YBX1. Approximately 70% of the top 500 genes that are positively correlated with ESR1 overlap with the top 500 genes that are negatively correlated with YBX1. (**E**) A Venn diagram showing the overlap of the top 500 genes that are negatively correlated with ESR1 and the top 500 genes that are positively correlated with YBX1. Approximately 64% of the top 500 genes that are negatively correlated with ESR1 overlap with the top 500 genes that are positively correlated with YBX1. (**F**) A Venn diagram showing the overlap of the top 500 genes that are negatively correlated with ESR1 and the top 500 genes that are negatively correlated with YBX1. The top 500 genes that are negatively correlated with ESR1 showed no overlap with the top 500 genes that are negatively correlated with YBX1.

Previous studies from many laboratories have consistently shown that the enhanced expression of YBX1 protein or mRNA in mammary tumors is significantly correlated with malignant progression or poor outcomes in patients with breast cancer [21, 23, 27, 28]. To identify the genes that are potentially regulated by YBX1, we evaluated RNA sequencing (RNA-seq) data from a cohort of 825 invasive breast cancer patients [29] obtained from The Cancer Genome Atlas (TCGA) (https://cancergenome.nih.gov/) [30, 31]. We first identified the top 500 genes that are positively or negatively correlated with YBX1 (Supplementary Tables 1–4). The top 20 genes that are significantly (*P* < 0.001) and positively correlated with YBX1 are listed in Figure 1A. Of these 20 genes, the enhanced expression of 8 (UQCRH, CTPS1, FMNL2, CDC20, B3GNT5, MTHFD1L, LRP8, and CDCA8) in tumors was found to be significantly associated with a poor prognosis for the patients (Figure 1A and Supplementary Figure 1A). The expression levels of all 8 of these genes were positively (*P* < 0.001) correlated with YBX1 expression levels (Supplementary Figure 1B). Furthermore, the top 20 genes that were found to be negatively correlated with YBX1 are listed in Figure 1B. ESR1 was found to be significantly and negatively correlated with YBX1, while the ER-coactivator genes FOXA1 and GATA3 were negatively correlated with YBX1 expression (Figure 1B). FOXA1 and GATA3 are involved in tumorigenesis and malignant progression in breast cancer cells in close correlation with the hormonal status [32, 33]. Of the top 20 negatively correlated genes, the enhanced expression of 14 (ESR1, CHCHD5, ATP5G2, GATA3, PCP2, THSD4, LRRC46, PEX11G, RUNDC1, ARSG, SIRT3, TBC1D9, ABAT, and EVL) in tumors was associated with a positive prognosis in patients (Figure 1B and Supplementary Figure 2A). The expression levels of all 14 of these genes were inversely (*P* < 0.001) correlated with YBX1 expression (Supplementary Figure 2B).

### Inverse association between genes correlated with YBX1 and those correlated with ESR1

The top 20 genes negatively correlated with YBX1 included many genes that are correlated with ESR1 and its effector genes, such as GATA3 and FOXA1 (Figure 1B). We next examined whether the genes correlated with YBX1 are associated with the genes correlated with ESR1. A Venn diagram revealed some overlap between the top 500 genes significantly correlated with YBX1 and those significantly correlated with ESR1 (Supplementary Tables 1–4). However, the top 500 genes positively correlated with ESR1 did not overlap with the top 500 genes positively correlated with YBX1 (Figure 1C and Supplementary Table 1). In contrast, 352 of the top 500 genes, including GATA3, FOXA1, SIRT3, MYB, and HDAC11, are common between the genes positively correlated with ESR1 and the genes negatively correlated with YBX1 (Figure 1D and Supplementary Table 2). Furthermore, 322 of the top 500 genes, including CDK6, UQCRH, and CTPS1, are common between the genes negatively correlated with ESR1 and the genes positively correlated with YBX1 (Figure 1E and Supplementary Table 3). However, there was no overlap between the top 500 genes that were negatively correlated with ESR1 and the top 500 genes that were negatively correlated with YBX1 (Figure 1F and Supplementary Table 4).

### Genes positively or negatively correlated with YBX1 predict the overall survival of patients

**Figure 2:**
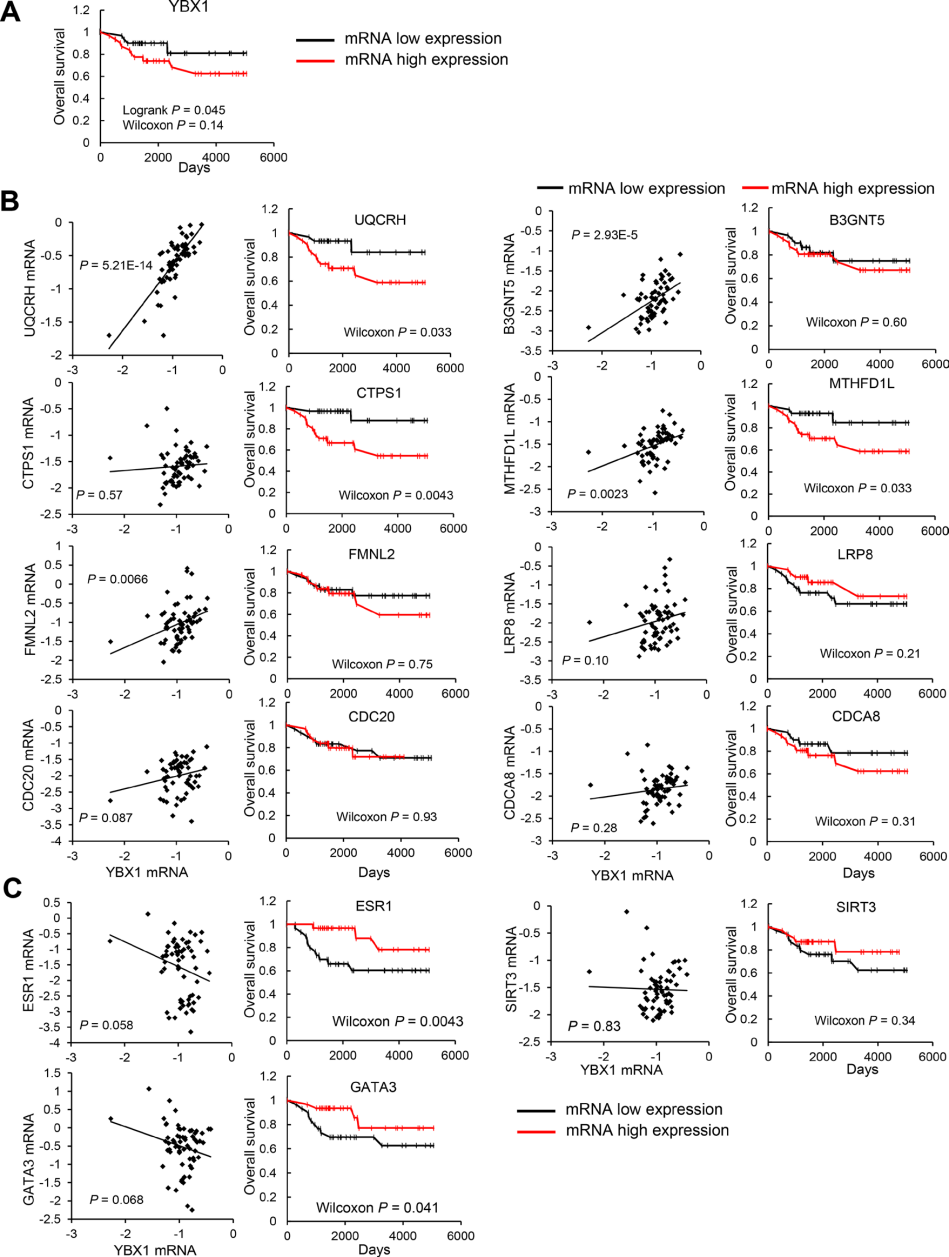
Genes correlated with YBX1 are closely associated with overall survival (**A**) The Kaplan-Meier overall survival analysis according to YBX1 mRNA expression in patients with breast cancer. High expression levels of YBX1 are associated with a poor prognosis in breast cancer patients. (**B**) Left: Correlation data for the YBX1 mRNA expression level versus the mRNA expression levels of genes positively correlated with YBX1. The statistical significance of the correlations was determined using the *χ*^2^ test. The linear regression curve is shown as a black line for significant correlations. Right: The Kaplan-Meier overall survival analysis according to the mRNA expression levels of genes positively correlated with the expression level of YBX1 in patients with breast cancer. (**C**) Left: Correlation data for the YBX1 mRNA expression level versus the mRNA expression levels of genes negatively correlated with YBX1. The statistical significance of the correlations was determined using the *χ*^2^ test. The linear regression curve is shown as a black line for significant correlations. Right: The Kaplan-Meier overall survival analysis according to the mRNA expression levels of genes negatively correlated with YBX1 in patients with breast cancer.

We further assessed whether genes positively or negatively correlated with YBX1 could predict the outcomes of breast cancer patients (*n* = 63) at Kyushu University Hospital. Patients with high expression levels of YBX1 in tumors had worse outcomes than those with low expression levels (Figure 2A). The expression levels of 8 genes selected from among the genes positively correlated with YBX1 were mostly positively correlated with YBX1 mRNA expression levels (Figure 2B). Patients with higher expression levels of UQCRH, MTHFD1L, and CTPS1 showed significantly unfavorable outcomes compared to those with low expression levels. In contrast, patients with higher expression levels of CDC20, B3GNT5, LRP8, CDCA8, and FMNL2 showed no significant difference in overall survival compared to those with lower expression levels (Figure 2B). Furthermore, the expression levels of ESR1, GATA3, and SIRT3, which are genes negatively correlated with YBX1, showed an inverse association with the expression level of YBX1; patients with high expression levels of ESR1 and GATA3 in tumors had better outcomes than those with low expression levels (Figure 2C).

**Figure 3:**
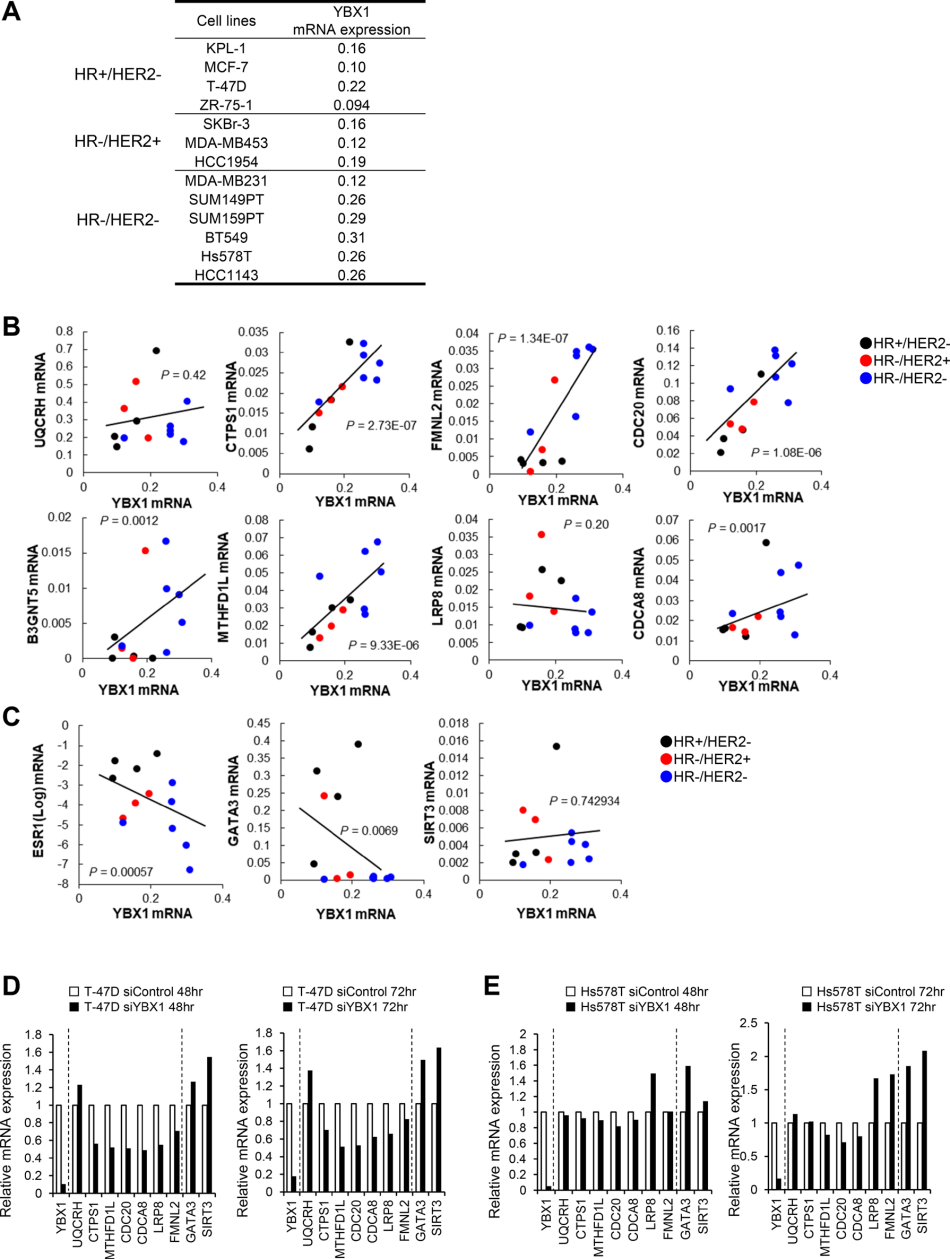
The close association of YBX1 and genes correlated with YBX1 in breast cancer cell lines (**A**) List of breast cancer cell lines and YBX1 mRNA levels in three subtypes of breast cancer cell lines. (**B**) Correlation data for the YBX1 mRNA expression level versus the mRNA expression levels of genes positively correlated with YBX1 in 13 breast cancer cell lines. The statistical significance of the correlations was determined using the *χ*^2^ test. The linear regression curve is shown as a black line for significant correlations. (**C**) Correlation data for the YBX1 mRNA expression level versus the mRNA expression levels of genes negatively correlated with YBX1 in 13 breast cancer cell lines. The statistical significance of the correlations was determined using the *χ*^2^ test. The linear regression curve is shown as a black line for significant correlations. (**D**, **E**) Quantitative RT-PCR showing relative mRNA expression levels of genes positively or negatively correlated with the expression level of YBX1 in T-47D (D) or Hs578T (E) cells treated with YBX1 siRNA (200 nM) for 48 h (left) or 72 h (right).

We next examined the association between YBX1 and the genes correlated with YBX1 in 13 breast cancer cell lines established from HR+/HER2-, HR-/HER2+, and HR-/HER2- tumors (Figure 3A). With the exception of UQCRH and LRP8, the expression levels of genes positively correlated with YBX1, such as CTPS1, FMNL2, CDC20, B3GNT5, MTHFD1L, and CDCA8, were significantly and positively correlated with the expression level of YBX1 in 13 breast cancer cell lines (Figure 3B). Furthermore, as shown in Figure 3C, the expression levels of ESR1 and GATA3 were inversely correlated with the expression level of YBX1, but there was no significant correlation between the expression levels of SIRT3 and YBX1.

We further examined the expression of 7 genes positively correlated with YBX1 and 2 genes negatively correlated with YBX1 when YBX1 expression was silenced in T-47D (HR+/HER2-) and Hs578T (HR-/HER2-) cells by siRNA (Figure 3D and 3E). The expression levels of 6 of the 7 genes that were positively correlated with the expression of YBX1 were reduced, and the expression levels of the 2 genes that were negatively correlated with the expression of YBXI were enhanced in YBX1-silenced T-47D cells (Figure 3D). The expression levels of 3 of the 7 genes that were positively correlated with the expression of YBX1 were also affected, and the expression levels of the two genes that were negatively correlated with the expression level of YBX1 were enhanced in YBX1-silenced Hs578T cells (Figure 3E).

### The expression of YBX1 and its correlated genes predicts recurrence susceptibility

**Figure 4:**
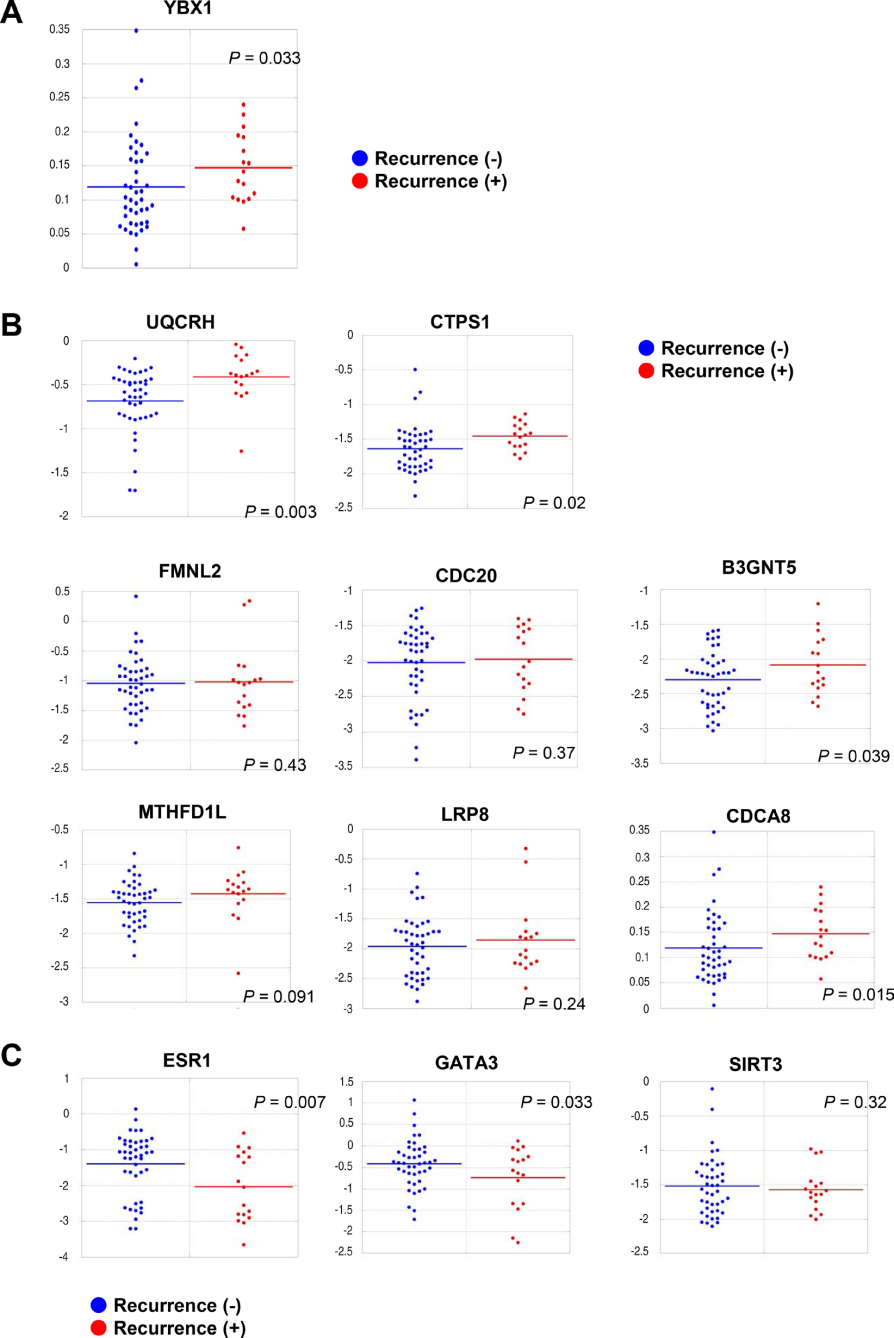
YBX1 is a predictive marker for recurrence and susceptibility to endocrine/chemotherapeutics (**A**) Dot plots showing the mRNA expression levels of YBX1 in the primary tumors of patients who relapsed after the initial surgical operation and in the primary tumors of patients who did not relapse. *P* value, two-sided Student’s *t*- test. (**B**) Dot plots showing the mRNA expression levels of genes positively correlated with the expression of YBX1 in the primary tumors of patients who relapsed after the initial surgical operation and in the primary tumors of patients who did not relapse. *P* value, two-sided Student’s *t*- test. (**C**) Dot plots showing the mRNA expression level of genes negatively correlated with the expression of YBX1 in the primary tumors of patients who relapsed after the initial surgical operation and in the primary tumors of patients who did not relapse. *P* value, two-sided Student’s *t*- test.

YBX1 promotes tumorigenesis and the malignant progression of breast cancer [21, 23, 27, 28]. Consistent with this notion, we explored whether YBX1 and the genes closely correlated with YBX1 were associated with breast cancer recurrence. The expression levels of YBX1 and its correlated genes were initially assessed when the tumors were surgically resected. The expression level of YBX1 in tumors was significantly (*P* = 0.033) higher in patients who relapsed after the initial surgical operation than in those who did not relapse (Figure 4A). Furthermore, the expression levels of 4 (UQCRH, CTPS1, B3GNT5, and CDCA8) of 8 genes positively correlated with YBX1 were significantly (*P* < 0.05) higher in recurrent tumors than in nonrecurrent tumors (Figure 4B). The expression levels of the other 4 genes (FMNL2, CDC20, MTHFD1L, and LRP8) were moderately higher in recurrent tumors than in nonrecurrent tumors. The expression levels of 2 of the 3 genes negatively correlated with YBX1 (ESR1 and GATA3) were significantly (*P* < 0.05) lower in patients who relapsed after their primary surgery than in those who did not relapse (Figure 4C), suggesting that YBX1 and its correlated genes could be used as predictive genes for recurrence and poor outcomes in breast cancer patients.

### Associations of YBX1 and ESR1 with overall survival by a principal component analysis

**Figure 5:**
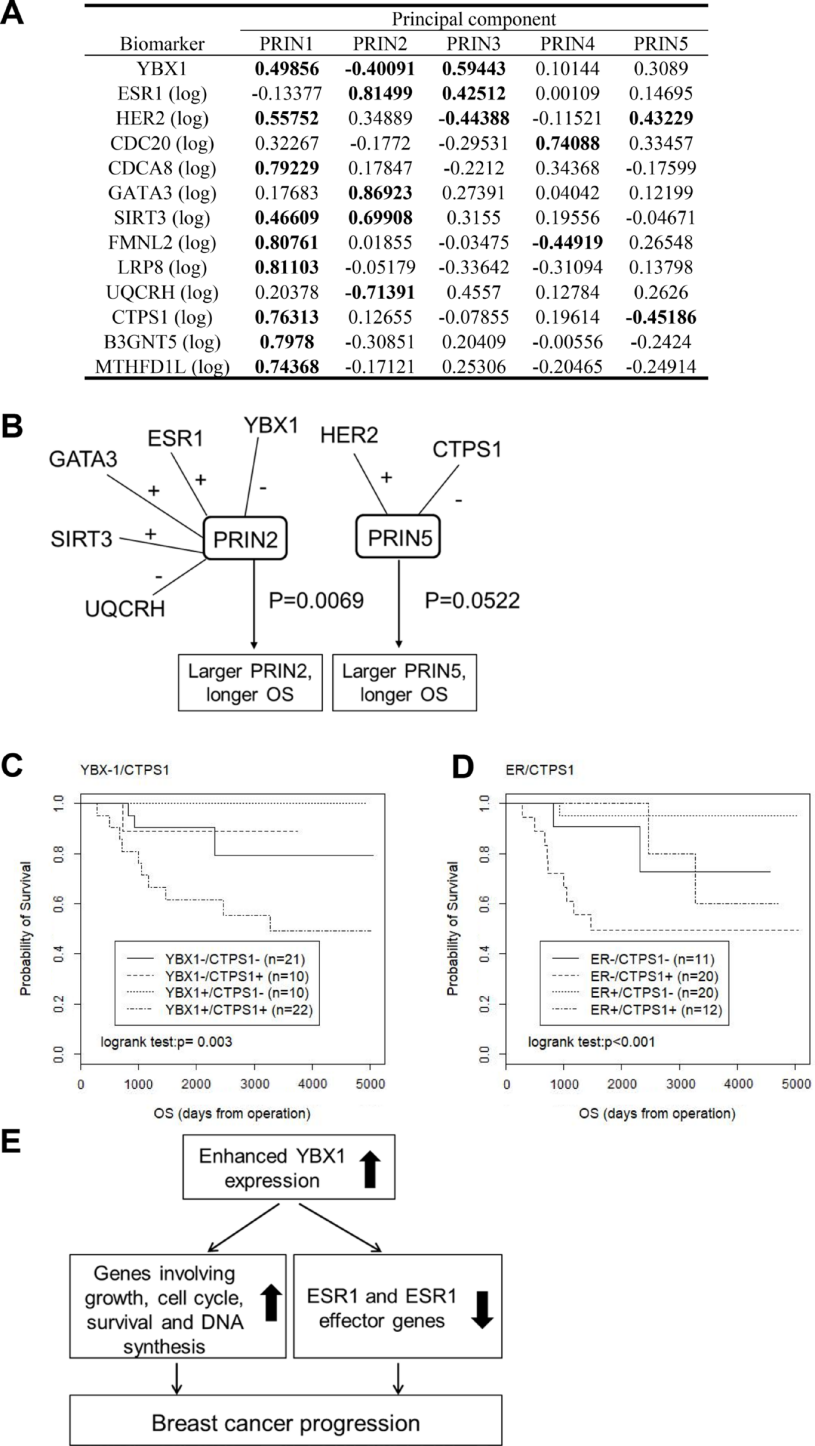
YBX1 and its correlated genes are predictive markers for a poor prognosis (**A**) Correlation coefficient between the principal components and biomarkers. (**B**) Association between the principal components and the overall survival; biomarkers with correlation coefficients > |0.4| with principal components were linked with the sign attached. (**C**) Kaplan-Meier plots for the four groups defined by the expression of YBX1 and CTPS1, where positivity (+) is defined as a biomarker value higher than its median. (**D**) Kaplan-Meier plots for the four groups defined by the expression of ESR1 and CTPS1, where positivity (+) is defined as a biomarker value higher than its median. (**E**) Our hypothetical model showing that YBX1 promotes the malignant progression of breast cancer in close correlation with the enhanced expression of its effector genes involved in cell growth, cell cycle, survival and DNA synthesis, as well as the decreased expression of ESR1 and various ESR1 effector genes.

The first 5 principal components constructed with the Kyushu University dataset (see Figure 2) had an 86% cumulative contribution ratio, so we restricted our attention to the first 5 principal components. Our previous studies demonstrated that HER2 is positively correlated with YBX1 in breast cancer [21, 26]. We thus added HER2 as a biomarker in addition to the 12 novel biomarkers in this analysis. Hereafter, the κ-th principal component is denoted by PRINκ. The correlation coefficients between the 5 principal components and the 13 biomarkers are presented in Figure 5A. We applied principal component Cox regression, in which the first 5 principal components were included as explanatory variables. The first principal component, PRIN1, was positively associated with most biomarkers, including YBX1 and HER2. PRIN1 may represent the general association among the 13 biomarkers. However, PRIN1 was found to be nonessential for determining the prognosis by principal component Cox regression, while PRIN2 and PRIN5 were significantly associated with overall survival (*P* = 0.0069 for PRIN2 and *P* = 0.0522 for PRIN5) (Figure 5B). PRIN2 was positively associated with ESR1, GATA3, and SIRT3 and negatively associated with YBX1 and UQCRH. PRIN5 was positively associated with HER2 and negatively associated with CTPS1. These results suggest that the two mechanisms induced by PRIN2 and PRIN5 play critical roles in the prognosis of breast cancer patients. To demonstrate the idea more simply, we represented PRIN2 by YBX1 or ESR1 and PRIN5 by CTPS1 and examined their influence on overall survival using a simple Kaplan-Meier analysis (Figure 5C and 5D). YBX1 and CTPS1 as well as ESR1 and CTPS1 were found to be independently associated with the prognosis, supporting the idea that PRIN2 and PRIN5 play important roles in the prognosis of breast cancer patients.

## DISCUSSION

The nuclear expression level of YBX1 and an increased total YBX1 expression level are predictive markers for poor prognosis in patients with breast cancer [19, 21, 23, 27, 28], ovarian cancer [34], prostate cancer [35], and other human malignancies [36]. Therefore, clarifying the precise mechanisms underlying the significant effect of YBX1 on prognosis will aid in the development of therapies for such cancers. In the present study, enhanced YBX1 expression was mostly correlated with enhanced expression of genes involved in cell growth, cell cycle, survival and DNA synthesis, as well as decreased expression of ESR1 and various ESR1 effector genes in breast cancer patients (Figures 1 and 2) and breast cancer cell lines (Figure 3). The increased or decreased expression levels of these genes correlated with the expression of YBX1 may thus limit the malignant progression of breast cancer (Figure 5E).

ER-targeted antiestrogens and other endocrine therapeutic drugs have greatly contributed to the improvement of breast cancer therapies [3, 4]. However, one serious problem is the emergence of tumors resistant to antiestrogen drugs during the treatment of ER-positive breast cancer patients. Our previous studies demonstrated a significant and reciprocal correlation between YBX1 and ER in breast cancers [21, 26], and showed that the enhanced expression of YBX1 markedly downregulates the expression of ESR1 and induces acquired resistance to antiestrogen therapeutics [26]. In the present study, more than 60% of the genes that were positively correlated with YBX1 were also negatively correlated with ESR1 (Figure 1E). Therefore, the enhanced expression of YBX1 is often reciprocally accompanied by the reduced expression of ER-dependent genes, which likely promotes breast cancer progression by driving ER-independent cell growth and survival (Figure 5E). Furthermore, we found that patients with higher expression of YBX1 showed poor outcome in ER-positive patients treated with anti-estrogen therapy (Supplementary Figure 3).

Oncotype DX and Mammaprint are frequently used in clinical practice for deciding the suitability of adjuvant chemotherapy for patients with ER-positive and node-negative breast cancer [37, 38]. Oncotype DX and Mammaprint involve reverse transcription polymerase chain reactions for the expression of 21 and 70 genes, respectively, using RNA derived from breast tumor tissues. ER-positive early-stage breast cancer patients can be classified with statistical significance into low- and high-risk groups by these assays. However, in those assays, more than 20 genes are analyzed, and the outcomes of ER-negative breast cancer cannot be predicted. In the present study, we demonstrated that patients with tumors expressing higher YBX1 mRNA levels experienced recurrence more often than those with lower mRNA levels (Figure 4A). Genes that were positively correlated with YBX1 showed a higher expression in recurrent tumors than in nonrecurrent tumors (Figure 4B). In contrast, two genes that were negatively correlated with YBX1 showed lower expression levels in recurrent tumors than in nonrecurrent tumors (Figure 4C). Furthermore, the enhanced expression levels of YBX1 and its correlated gene CTPS1 were able to predict poor outcomes of breast cancer (Figure 5C). Determining the expression levels of YBX1 and its correlated genes can therefore aid in the identification of patients with a high risk of recurrence, and YBX1 and its correlated genes may be useful biomarkers for the precise prediction of breast cancer patients with a high risk of recurrence.

Our and other research groups previously reported that phosphorylation of YBX1 by various kinases including AKT, S6K and RSK through receptor tyrosine kinase and integrin linked kinase induces nuclear translocation of YBX1 in close context with transcriptional activation of various genes including drug resistance and tumor growth related genes [39–42]. Development of therapeutic drugs by targeting YBX1 activation process could contribute to overcome breast cancer through inhibiting expression of predictive genes in present study.

Our present study indicates that the expression of YBX1 and its correlated genes could be used to predict not only poor outcomes but also resistance to endocrine therapeutics and chemotherapeutics in patients with breast cancer. Further follow-up studies in a larger number of patients would be required to confirm our present findings. Targeting YBX1 is expected to help further improve the utility of precision medicine for breast cancer.

## MATERIALS AND METHODS

### Cell culture

The human breast cancer cell lines MCF-7, T-47D, SKBr-3, MDA-MB231, MDA-MB453, ZR-75-1, HCC1954, BT549, Hs578T, and HCC1143 were purchased from the American Type Culture Collection (Manassas, VA, USA). SUM159PT and SUM149PT cells were purchased from Asterand (Detroit, MI, USA). KPL-1 was purchased from Health Science Research Resources Bank (Osaka, Japan). KPL-1, MCF-7, T-47D, SKBr-3, MDA-MB231, MDA-MB453, and Hs578T cells were cultured at 37°C in DMEM supplemented with 10% fetal bovine serum (FBS) in a humidified atmosphere containing 5% CO_2_. SUM159PT and SUM149PT cells were maintained with Ham’s F-12 nutrient mixture containing 5% FBS, 5 μg/mL insulin, 1 μg/mL hydrocortisone, and 10 mmol/L HEPES. ZR-75-1, HCC1954, and HCC1143 cells were cultured with RPMI containing 10% FBS. BT549 cells were cultured with RPMI containing 10% FBS and 0.8 μg/mL insulin. All cell lines were passaged for ≤ 6 months and were not further tested or authenticated by the authors.

### Transfection of small interfering RNA

The siRNA corresponding to the nucleotide sequence of YBX1 (siYBX1;5′-GGUUCCCACCUUACUACAU-3′) was purchased from QIAGEN Inc. (Valencia, CA, USA). Cells were transfected with siRNA duplexes using Lipofectamine RNAiMAX and Opti-MEM (Invitrogen) according to the manufacturer’s recommendations.

### Patient information

Breast cancer tissue specimens were obtained from 64 Japanese patients who underwent surgery without neoadjuvant systemic therapy at the Department of Surgery and Science, Kyushu University Hospital, between 2004 and 2014. The study was approved by the institutional review board of the university (30–40). Immediately after surgery, the specimens for extraction of the total RNA were placed in liquid nitrogen and stored at −80°C. The clinical data were obtained from the patients’ medical records. Endocrine therapy consisted of an aromatase inhibitor, either tamoxifen or toremifene for postmenopausal women, and an LH-RH agonist for premenopausal women. Chemotherapy regimens, including epirubicin and cyclophosphamide, 5-fluorouracil (5-FU), cyclophosphamide, methotrexate and 5-FU and taxanes, were administered based on the clinicopathological findings. The median follow-up was 2678 days. An overview of the clinical information regarding the patients’ age, sex, pathological diagnosis of the primary tumor, status of recurrence, stage, adjuvant therapy, and immunohistochemistry scores of ER, PGR, and HER2 are presented in Supplementary Table 5. To avoid confusion with the TCGA dataset, we have referred to these data as the Kyusyu University dataset in this paper.

### Quantitative reverse transcription polymerase chain reaction (qRT-PCR)

Total RNA was isolated from human breast tumor tissue using ISOGEN (Nippon Gene Co., Ltd., Tokyo, Japan) according to the manufacturer’s instructions. The RNA concentration was assessed by spectrophotometry at 260 nm. qRT-PCR was performed using the Real-Time PCR system 7300 (Applied Biosystems, Foster City, CA, USA). In these analyses, the average expression levels of genes correlated with YBX1 were calculated, and the patients were then classified into high- and low-mRNA expression groups using the average as a cut-off point. Survival curves were plotted using the Kaplan-Meier method and compared using the log-rank test or Wilcoxon’s test. Survival data were evaluated using a multivariate Cox proportional hazards model. Genes were evaluated as having either a poor prognostic ability (*P* < 0.05) or a good prognostic ability (*P* < 0.05).

### Statistical analyses

In this manuscript, we used two clinical datasets. The first dataset was from a cohort of 825 invasive breast cancer patients obtained from TCGA (the cBioPortal for Cancer Genomics; http://www.cbioportal.org). Using Spearman’s rank correlation coefficient, we confirmed the negative association between ESR1 and YBX1. In addition, by ranking genes in order of Spearman’s rank correlation coefficients for the associations between YBX1 and those genes (Figure 1A and 1B), the importance of the association between ESR1 and YBX1 was investigated. The associations between the expression levels of YBX1 and its correlated genes were assessed using *χ*^2^ tests. The second dataset was the Kyushu University dataset, the details of which are described in the patient information section above. Using these data, we examined the associations among the mRNA levels of 13 biomarkers and their influence on overall survival, which was defined as the duration from surgery to death due to any cause and could be right-censored at the date of last follow-up. To this end, we employed a principal component Cox regression analysis. To avoid biases due to influential observations, we assessed the skewness of the mRNA levels of each biomarker, and the mRNA levels of biomarkers other than YBX1 were log-transformed. Regarding the principal components as underlying independent mechanisms behind the 13 biomarkers (possibly pathways), we tried to speculate about the underlying mechanism in breast cancer patients. Using principal component Cox regression, the associations between the underlying mechanisms and prognosis were investigated. To simplify interpretation, we focused on the principal components attaining an 80% cumulative contribution ratio, which was interpreted as 80% variation of the 13 biomarkers being explained by these principal components.

## SUPPLEMENTARY MATERIALS FIGURES AND TABLES










