# Supplementary material for: Y-box binding protein YBX1 and its correlated genes as biomarkers for poor outcomes in patients with breast cancer
Source: Oncotarget. 2018 Dec 14;9(98):37216–28. doi: 10.18632/oncotarget.26469 (PMC6324687; doi:10.18632/oncotarget.26469)
Supplement: Supplementary file 1 [file oncotarget-09-37216-s001.pdf]

Y-box binding protein YBX1 and its correlated genes as biomarkers for poor outcomes in patients with breast cancer

SUPPLEMENTARY MATERIALS

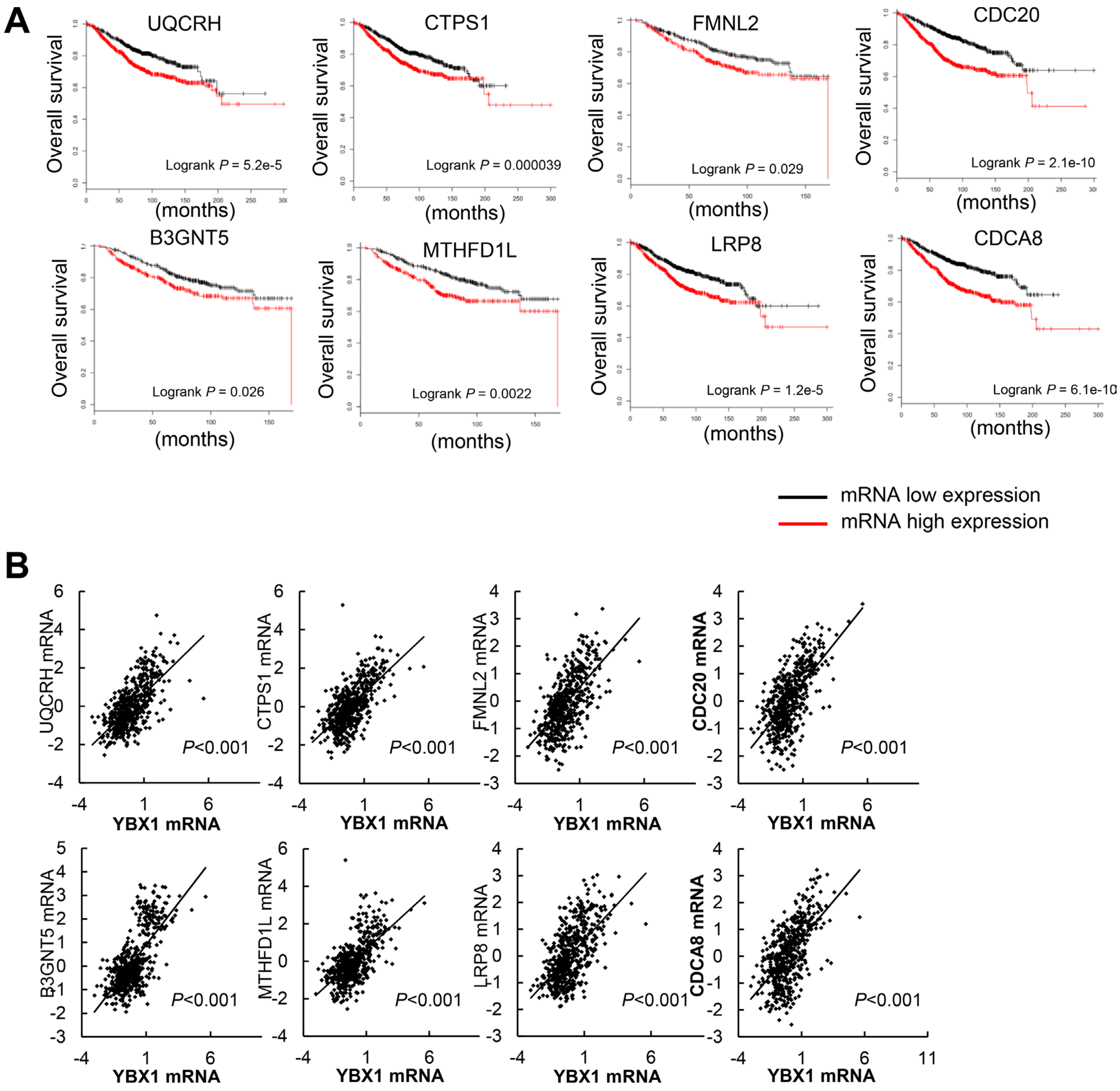

Supplementary Figure 1: YBX1 positively correlated with poor prognostic factors. (**A**) Kaplan-Meier overall survival according to UQCRH, CTPS1, FMNL2, CDC20, B3GNT5, MTHFD1L, LRP8 and CDCA8 expression in patients with invasive breast cancer. High expression of these genes is associated with poor prognosis of invasive breast cancer patients. (**B**) Correlation data for YBX1 versus UQCRH, CTPS1, FMNL2, CDC20, B3GNT5, MTHFD1L, LRP8 and CDCA8 mRNAs expression. The statistical significance of the correlations was determined using X^2 test. The linear regression curve is shown as a black line that indicates significant ($P < 0.001$) correlations.

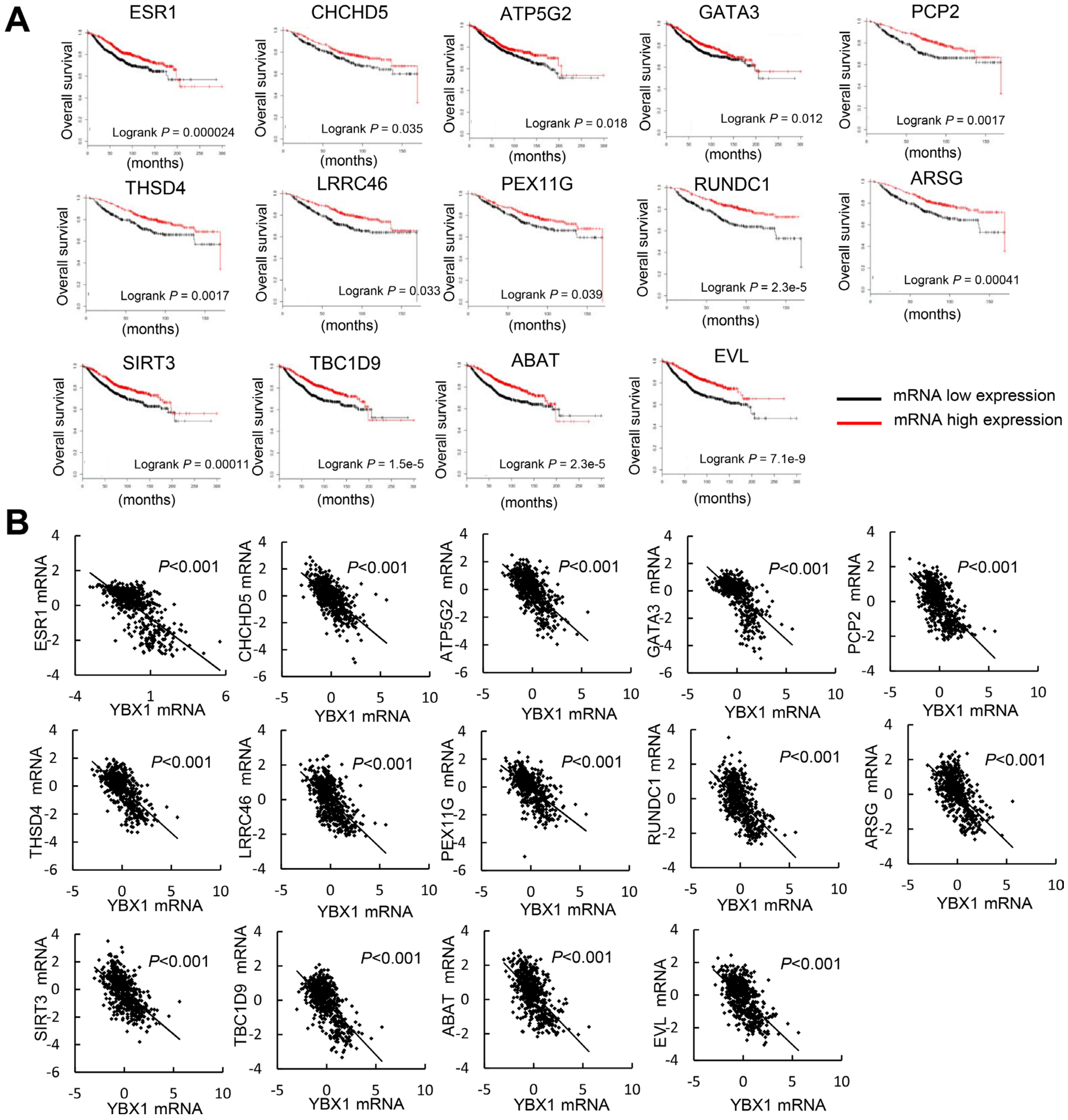

Supplementary Figure 2: YBX1 negatively correlated with good prognostic factors. (**A**) Kaplan-Meier overall survival according to ESR1, CHCHD5, ATP5G2, GATA3, PCP2, THSD4, LRRC46, PEX11G, RUNDC1, ARSG, SIRT3, TBC1D9, ABAT and EVL expression in patients with invasive breast cancer. High expression of these genes is associated with good prognosis of invasive breast cancer patients. (**B**) Correlation data for YBX1 versus ESR1, CHCHD5, ATP5G2, GATA3, PCP2, THSD4, LRRC46, PEX11G, RUNDC1, ARSG, SIRT3, TBC1D9, ABAT and EVL mRNAs expression. The statistical significance of the correlations was determined using X^2 test. The linear regression curve is shown as a black line that indicates significant ($P < 0.001$) correlations.

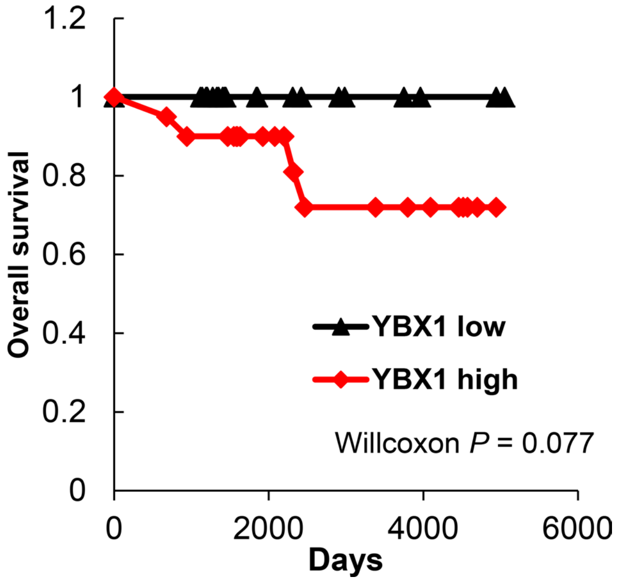

Supplementary Figure 3: YBX1 is predictive biomarker for ER-positive breast cancer patients. Kaplan-Meier overall survival according to YBX1 mRNA expression in ER-positive patients treated with endocrine therapy.

Supplementary Table 1: Association of YBX1 positively correlated genes and ESR1 positively correlated genes. See Supplementary_Table_1

Supplementary Table 2: Association of YBX1 negaatively correlated genes and ESR1 positively correlated genes. See Supplementary_Table_2

Supplementary Table 3: Association of YBX1 positvely correlated genes and ESR1 negaatively correlated genes. See Supplementary_Table_3

Supplementary Table 4: Association of YBX1 negaatively correlated genes and ESR1 negaatively correlated genes. See Supplementary_Table_4

Supplementary Table 5: Patients background

	ER(IHC)	PgR(IHC)	HER2	Recurrence	Overall Survival Status	Survival periods (Days)	Stage	Adjuvant chemotherapy	Adjuvant Endocrine therapy
1	0	0	1	1	LIVING	5055	3	No	No
2	1	1	0	0	LIVING	5052	3	Yes	Yes
3	0	0	1	1	DECEASED	1061	3	Yes	No
4	0	0	1	0	LIVING	4955	2	Yes	No
5	1	1	1	0	LIVING	4943	2	No	Yes
6	1	1	1	0	LIVING	4943	2	Yes	Yes
7	0	0	1	1	DECEASED	1006	2	No	No
8	1	1	0	1	DECEASED	3270	3	Yes	
9	0	0	0	1	DECEASED	503	3	Yes	No
10	0	0	1	0	LIVING	4766	2	Yes	No
11	1	0	0	1	DECEASED	2467	3	Yes	Yes
12	1	0	1	0	LIVING	4698	3	Yes	Yes
13	1	1	0	0	LIVING	4572	2	No	Yes
14	1	1	0	1	LIVING	4516	2	No	Yes
15	0	1	0	0	LIVING	4458	2	Yes	Yes
16	0	0	0	1	DECEASED	295	3	Yes	
17	1	1		1	DECEASED	678	2	No	Yes
18	0	0	0	1	DECEASED	725	2	Yes	No
19	1	1		0	LIVING	4094	3	Yes	Yes
20	1	1	0	0	LIVING	3963	2	No	Yes
21	0	0	0	0	LIVING	3961	2	No	No
22	1	1	0	1	DECEASED	2319	2	No	Yes
23	1	1	0	0	LIVING	3799	2	No	Yes
24	1	1	0	0	LIVING	3750	3	Yes	Yes
25	0	0	1	0	LIVING	3473	2	Yes	
26	1	1	0	0	LIVING	3382	3	No	Yes
27	1	1	0	1	DECEASED	941	2	No	Yes
28	0	0	1	1	DECEASED	825	2	No	No
29	1	1	0	0	LIVING	2983	2	No	Yes
30	1	1	0	0	LIVING	2906	3	Yes	Yes
31	0	0	1	1	DECEASED	1476	2	Yes	No
32	1	1	0	1	LIVING	2421	2	Yes	Yes
33	0	0	1	0	LIVING	2372	2	No	No
34	1	1	1	0	LIVING	2311	2	Yes	Yes
35	1	1	0	0	LIVING	2199	3	Yes	Yes
36	0	0	0	0	LIVING	2173	2	Yes	No
37	1	1	1	0	LIVING	2080	3	Yes	Yes
38	0	0	1	0	LIVING	2029	2	Yes	No
39	0	0	0	1	DECEASED	1172	2	Yes	No
40	1	1	0	0	LIVING	1924	2	No	Yes
41	1	1	0	0	LIVING	1854	2	Yes	Yes
42	1	1	1	0	LIVING	1842	2	Yes	Yes

43	0	0	1	0	LIVING	1833	3	Yes	No
44	0	0	0	0	LIVING	1744	2	Yes	No
45	1	1	0	0	LIVING	1632	3	Yes	Yes
46	1	1	0	0	LIVING	1595	2	No	Yes
47	1	1	0	0	LIVING	1583	2	No	Yes
48	1	1	0	0	LIVING	1548	2	Yes	Yes
49	1	1	0	0	LIVING	1471	2	Yes	Yes
50	1	1	0		LIVING	1464	3	No	No
51	1	1	0	0	LIVING	1443	2	No	Yes
52	1	1	1	0	LIVING	1429	2	Yes	Yes
53	1	1	0	0	LIVING	1406	3	Yes	Yes
54	1	0		0	LIVING	1352	2	No	Yes
55	1(AB), 1(AC)	0(AB), 1(AC)	1(AB), 0(AC)	0	LIVING	1329	2	No	Yes
56	1	1	0	0	LIVING	1322	3	Yes	Yes
57	1	1	0	1	LIVING	1275	3	Yes	Yes
58	1	1	1	0	LIVING	1205	2	Yes	Yes
59	1	0	0				2	No	Yes
60	1	1	0	0	LIVING	1191	2	No	Yes
61	0	0	0	0	DECEASED	738	3	Yes	No
62	1	1	0	0	LIVING	1140	3	Yes	Yes
63	1	1	0	0	LIVING	1119	2	No	Yes
64	0	0	0				3	No	No
